# Rapid Identification of Berberine Metabolites in Rat Plasma by UHPLC-Q-TOF-MS

**DOI:** 10.3390/molecules24101994

**Published:** 2019-05-24

**Authors:** Peng Xu, Chen Xu, Xiaoxia Li, Dan Li, Yan Li, Jiebing Jiang, Ping Yang, Gengli Duan

**Affiliations:** 1 Department of Pharmaceutical Analysis, School of Pharmacy, Fudan University, Shanghai 201203, China; 12211030029@fudan.edu.cn (P.X.); chenxu@fudan.edu.cn (C.X.); yanli@fudan.edu.cn (Y.L.); 15111030029@fudan.edu.cn (J.J.); 2 Department of Pharmacology, School of Pharmacy, Fudan University, Shanghai 201203, China; 15002195516@163.com (X.L.); 14211030035@fudan.edu.cn (D.L.); 3 Instrumental Analysis Center, School of Pharmacy, Fudan University, Shanghai 201203, China

**Keywords:** berberine, metabolite identification, high resolution mass spectrometry, rat plasma

## Abstract

In this study, a reliable and rapid method based on ultra high performance liquid chromatography combined with quadrupole time-of-flight tandem mass spectrometry (UHPLC-Q-TOF-MS) technology and MetabolitePilot^MT^ software was developed for berberine metabolites identification in rat plasma. The chemical structures of the metabolites and their product ions were tentatively characterized or identified according to the molecular weights detected and MS/MS data. In all, nine metabolites, including M1 (demethyleneberberine, C_19_H_18_NO_4_, *m/z* 324), M2 (glucuronic acid-conjugated demethyleneberberine, C_25_H_26_NO_10_, *m/z* 500), M3 (diglucuronide-conjugated demethyleneberberine, C_31_H_34_NO_16_, *m/z* 676), M4 (glucuronic acid-conjugated jatrorrhizine or glucuronic acid-conjugated columbamine, C_26_H_28_NO_10_, *m/z* 514), M5 (berberrubine or thalifendine, C_19_H_16_NO_4_, *m/z* 322), M6 (glucuronic acid-conjugated berberrubine or glucuronic acid-conjugated thalifendine, C_25_H_24_NO_10_, *m/z* 498), M7 (sulfite-conjugated berberrubine or sulfite-conjugated thalifendine, C_19_H_16_NO_7_S, *m/z* 402), M8 (dihydroxy berberrubine or dihydroxy thalifendine, C_19_H_16_NO_6_, *m/z* 354) and M9 (dihydroxy berberine, C_20_H_18_NO_6_, *m/z* 368) were tentatively characterized or identified. Several new deposition patterns and three new metabolites (M7, M8 and M9) are reported in this paper for the first time. This work not only provides significant insights into the understanding of the metabolic pathways of berberine, but also contributes in identifying potential active drug candidates from the metabolites.

## 1. Introduction

**Figure 1:**
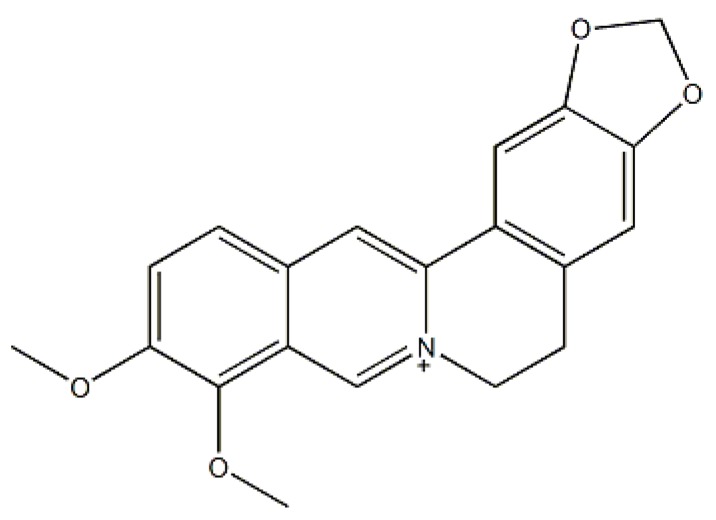
Molecular structure of berberine.

Berberine is an isoquinoline alkaloid extracted from traditional Chinese medicine such as *Berberis aristata*, Cortex Phellodendri and Coptidis rhizoma. Its chemical structure is shown in Figure 1. Berberine has been used as chloride salt form for treatment of secretory diarrhea and gastroenteritis in China for many years. It shows various pharmacological properties, such as antimicrobial [1], antidiarrheic [2], antiarrhythmic [3], anti-inflammatory [4] and anticancer actions [5]. In addition, it can decrease both cholesterol [6] and serum triglycerides [7]. The plasma concentration of berberine is very low after oral administration because berberine is poorly absorbed and extensively metabolized in the body [8]. So, the metabolites of berberine are the main existing forms in the body, which would account for the many different pharmacological effects of berberine in vivo [9]. So many pharmacological properties may be derived from different metabolites which may generated by many different metabolic pathways. Thus, making clear the berberine metabolic pathways is of significant importance for elucidating its pharmacological mechanisms.

So far, some studies have been set up to determine berberine metabolites. Ma et al. have concentrated on metabolites in rats feces, urine, and bile after administration of berberine [10]. Pan et al. put their efforts on the characterization and identification of metabolites in urine of berberine of healthy subjects [11], while Tsai et al. stressed the metabolites of berberine in the bile of rats [12]. In previous studies on the metabolism of berberine only some known metabolites such as demethyleneberberine, berberrubine, thalifendine, jatrorrhizine and their glucuronide conjugates were consistently identified [13,14]. The data remained stagnant and could not clearly illustrate the actual metabolism of berberine. Furthermore, the metabolites of berberine were detected by LC-MS or NMR spectroscopy in these previous studies, which might result in the neglect of some low-abundance metabolites. Pharmacokinetic studies have suggested berberine has low bioavailability [8]. The concentration of berberine in plasma is very low after oral administration because the absorption of berberine is poor [15]. Thus, extremely low concentrations of berberine in plasma increase the difficulty of the analysis of its metabolites.

UHPLC-Q-TOF-MS has become a reliable and powerful analytical instrument used for the identification of drug metabolites in complex biological matrix [16,17]. In our work, a rapid, sensitive and high resolution UHPLC-Q-TOF-MS method was established to identify berberine metabolites in rat plasma, resulting in that even extremely low concentrations of metabolites of berberine could be detected. By this method nine metabolites of berberine were simultaneously identified in rat plasma samples. Furthermore, three metabolites have not been reported before, which contributes to the finding of new metabolic pathways of berberine. Finally, the metabolism of berberine was elucidated comprehensively. This work not only provides significant insights into the understanding of the metabolic pathways of berberine, but also contributes in identifying potential active drug candidates from the metabolites.

## 2. Results and Discussion

### 2.1. Optimization of Detection Conditions

**Figure 2:**
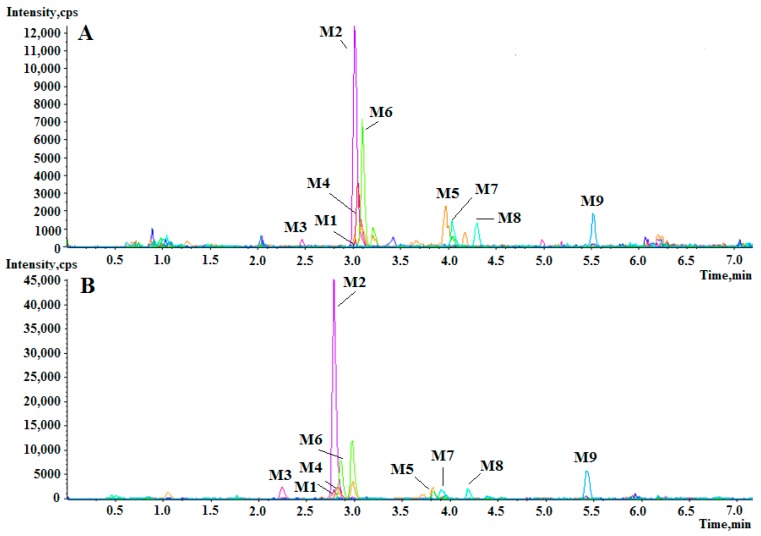
The extracted ion chromatogram of berberine metabolites samples. Formic acid (0.05%) in mobile phases (**A**) and 0.1% formic acid in mobile phases (**B**).

In order to improve detection efficiency, several conditions were optimized. In the step of sample preparation, the type of redissolved solvent had obvious influence on the detection result. Considering that the berberine metabolites belong to strongly polar compounds, 20% ACN was used as redissolved solvent. UPLC parameters, especially mobile phases, were examined. Berberine is a type of isoquinoline alkaloid and the majority of its metabolites have similar structure. Formic acid can help the ionization of alkaloids in the positive ion mode and promote the sharp shape of the peaks, both of which contribute to the increased signal abundance of the metabolites. The results suggested that 0.1% formic acid in mobile phases could increase the metabolites signal abundance compared to 0.05% formic acid in mobile phases (Figure 2). So the optimal mobile phase solution was composed of 0.1% formic acid, which was chosen for LC-MS analysis.

A small injection volume of 5 μL was tried before, but the intensities of some metabolites such as M7, M8 and M9 were too low to be detected. In order to improve the intensities of some low abundance metabolites, the injection volume was set at 20 μL. Then the separation of analytes was acceptable with the help of high resolution mass spectrometry detection. Before identifying the potential metabolites, the mass spectral behavior of the berberine should be comprehensively investigated and understood. In our preliminary study, positive and negative ion modes were compared to find the best experimental conditions. As the abundance of the product ions were more abundance in the positive ion mode, the positive ion mode was chosen for MS detection. Furthermore, the source temperature was also tested in order to improve the intensity of the response. When the temperature increased to 550 °C, the mass spectral response of berberine reached a steady level. Therefore, a final source temperature of 550 °C was chosen to perform the experiments.

### 2.2. Identification of Berberine Metabolites in Rat Plasma

**Figure 3:**
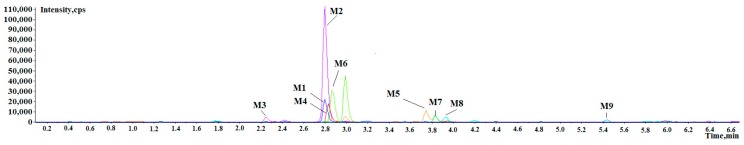
The extracted ion chromatogram of the berberine metabolites M1–M9 in rat plasma.

**Figure 4:**
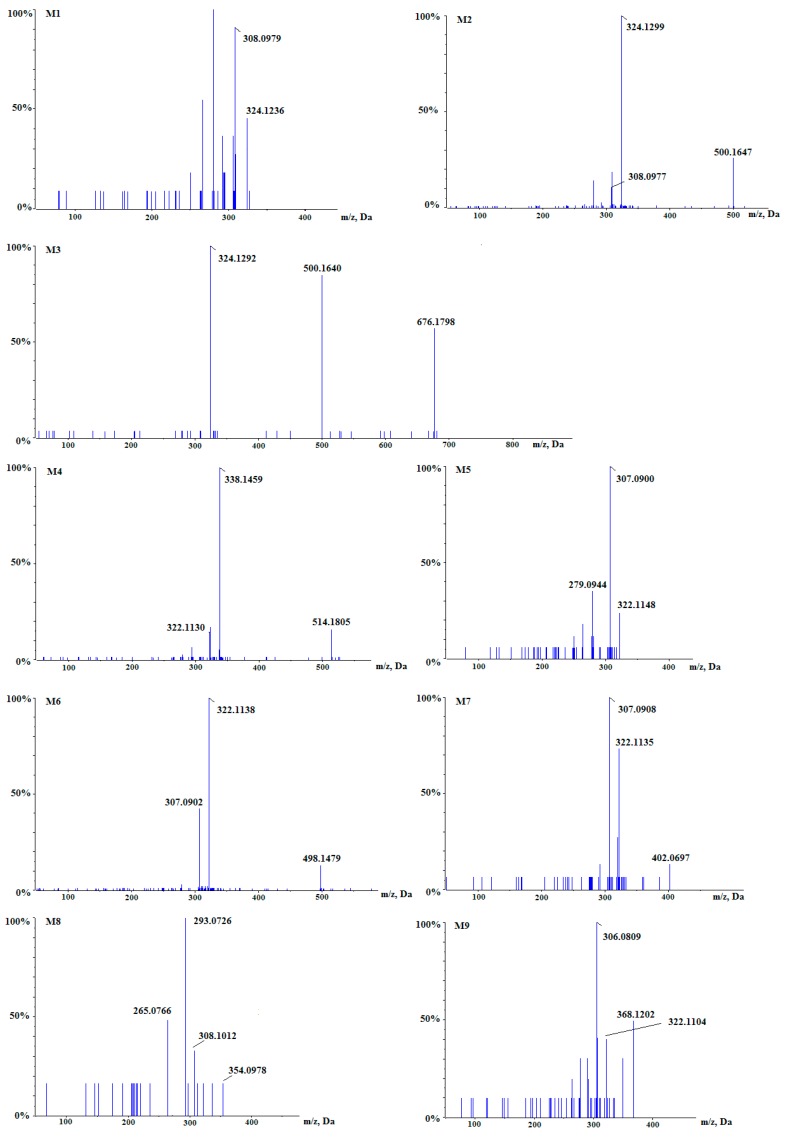
The MS^2^ spectra of berberine metabolites M1–M9 in rat plasma.

Berberine and its metabolites in rat plasma were analyzed by a rapid, sensitive and high resolution UHPLC-Q-TOF-MS method, and further identified with the help of MetabolitePilot^TM^ 1.5 software. As shown in Figure 3, nine berberine metabolites M1–M9 were detected. Their detailed information, including formulas, retention times, accurate molecular masses and fragment ions, is listed in Table 1. In addition, the MS^2^ spectra of berberine metabolites M1–M9 are shown in Figure 4.

Metabolite M1, with [M]+ of *m/z* 324.1236 (Figure 4), was eluted at 2.79 min, which was 12 Da less than berberine. The mass weight results suggested that the molecular formula of C_19_H_18_NO_4_. By losing CH_4_ the product ion at *m/z* 308.0979 was produced. M1 was preliminarily proposed to be demethyleneberberine.

Metabolite M2, with [M]+ of *m/z* 500.1647 (Figure 4), was eluted at 2.78 min, 176 Da higher than M1 (demethyleneberberine), indicating that M2 was the glucuronide conjugate of M1 (demethyleneberberine). The molecular formula of M2 was C_25_H_26_NO_10_ suggested by the mass weight measurement. By neutral losing of 176 Da from the [M]+ ion, the major product ion at *m/z* 324.1299 was produced. And the fragmentation patterns of M2, with *m/z* 308.0977 by losing CH_4_ from *m/z* 324.1299, were analogous with M1 (demethyleneberberine), identifying M2 as the glucuronide-conjugated demethyleneberberine.

Metabolite M3 which was 176 Da higher than M2, produced a precursor ion at *m/z* 676.1978 (Figure 4) and it was eluted at 2.23 min. The mass weight results indicated the molecular formula of C_31_H_34_NO_16_. The fragment ion at *m/z* 500.1640 was produced by the loss of a glucuronide group. Similar to M2, *m/z* 324.1292 was formed by loss of two glucuronide groups, identifying M3 as diglucuronide-conjugated demethyleneberberine.

Metabolite M4, which was 178 Da higher than berberine, yielded a precursor ion at *m/z* 514.1805 (Figure 4) and it was eluted at 2.81 min. It was demonstrated that the molecular formula was C_26_H_28_NO_10_ by the mass weight measurement. The product ion at *m/z* 338.1459 was produced by the loss of a glucuronide group to form the corresponding aglycon (jatrorrhizine or columbamine). The characteristic product ion at *m/z* 322.1130 was produced by losing CH_4_. M4 was preliminarily proposed to be glucuronide-conjugated jatrorrhizine or columbamine.

Metabolite M5, which was 14 Da less than berberine, yielded [M]+ ion at *m/z* 322.1148 (Figure 4) and produced *m/z* 307.0900 by losing CH_3_ and the *m/z* 279.0944 [M − CH3 − CO]^+^ by further loss of CO. It was demonstrated that the molecular formula was C_19_H_16_NO_4_ by the mass weight measurement. M5 was preliminarily proposed to be berberrubine or thalifendine.

Metabolite M6 (C_25_H_24_NO_10_) yielded [M]+ peak at *m/z* 498.1479 (Figure 4) and it was eluted at a 2.85 min, which was 176 Da more than the M5 (berberrubine or thalifendine, C_19_H_16_NO_4_), indicating that M6 was berberrubine glucuronide or thalifendine glucuronide. M6 produced product ions at *m/z* 322.1138 and *m/z* 307.0902. The product ion *m/z* 322.1138 [M-C_6_H_8_O_6_]+ was formed by loss of the glycoside moiety to form the corresponding aglycon (berberrubine or thalifendine). The product ion *m/z* 307.0902 [M-C_6_H_8_O_6_-CH_3_]+ was produced by losing CH_3_ from *m/z* 322.1138 [M-C_6_H_8_O_6_]+.

Metabolite M7 yielded [M]+ peak at *m/z* 402.0697 (Figure 4) and it was eluted at 3.82 min, which was 80 Da higher than the M5 (berberrubine or thalifendine, C_19_H_16_NO_4_), suggesting that M7 was the sulfite conjugate of M5 (berberrubine or thalifendine). The molecular formula was demonstrated as C_19_H_16_NO_7_S. The character fragment at *m/z* 322.1135 was produced by losing SO_3_ and *m/z* 307.0908 was formed by further losing CH_3_. So M7 was the sulfite conjugate of berberrubine or sulfite conjugate of thalifendine.

Metabolite M8 yielded a [M]^+^ at *m/z* 354.0978 (Figure 4) and was eluted at 3.90 min, which was 32 Da more than M5 (berberrubine or thalifendine), indicating that dihydroxylation happened in the disposition process. The derived element composition was C_19_H_16_NO_6_. Precursor ion produced the product ion at *m/z* 308.1012 by losing CH_2_O_2_. Product ion *m/z* 308.1012 further formed ion at *m/z* 293.0726 by loss of CH_3_. Therefore M8 was tentatively identified as dihydroxy berberrubine or thalifendine.

Metabolite M9 produced a precursor ion at *m/z* 368.1202 (Figure 4), 32 Da higher than berberine and it was eluted at 5.43 min, indicating that dihydroxylation happened during the disposition course. It was demonstrated that the molecular formula was C_20_H_18_NO_6_ by the mass weight measurement. The product ion at *m/z* 322.1104 was produced by loss of a CH_2_O_2_ and the product ion at *m/z* 306.0809 was formed by further loss of an O. M9 was preliminarily proposed to be dihydroxy berberine.

By comparing with previously published articles concerning with the identification of the metabolites of berberine, the validity of the identifications of this research was proved. Ma et al. also found the metabolites demethyleneberberine (M1, C_19_H_18_NO_4_, *m/z* 324), glucuronic acid-conjugated demethyleneberberine (M2, C_25_H_26_NO_10_, *m/z* 500) and diglucuronide-conjugated demethyleneberberine (M3, C_31_H_34_NO_16_, *m/z* 676) in rat urine with the product ions *m/z* at 308, 324 and 500, which were the same as the product ions found in the present research [10]. Qiu et al. isolated and identified the urinary metabolites glucuronic acid-conjugated jatrorrhizine or columbamine (M4, C_26_H_28_NO_10_, *m/z* 514) of berberine in human with typical product ion of *m/z* 338, which was consistent with the findings in this paper [18]. It has been reported that berberrubine or thalifendine (M5, C_19_H_16_NO_4_, *m/z* 322) and glucuronic acid-conjugated berberrubine or thalifendine (M6, C_25_H_24_NO_10_, *m/z* 498) were detected in rat plasma. The characteristic fragment ions of them were at *m/z* 279, 307 and 322, which were also consistent with the results in this paper [19]. As for sulfite-conjugated berberrubine or sulfite-conjugated thalifendine (M7, C_19_H_16_NO_7_S, *m/z* 402), dihydroxy berberrubine or dihydroxy thalifendine (M8, C_19_H_16_NO_6_, *m/z* 354) and dihydroxy berberine (M9, C_20_H_18_NO_6_, *m/z* 368), they were reported for the first time in this paper, and were tentatively identified by their fragment ions and fragment patterns.

### 2.3. Proposed Metabolic Pathway of Berberine in Rats

Pharmacokinetic studies have revealed that berberine shows a poor oral bio-availability [8], but it has still exhibited commendable biological activities. Papers indicated that the metabolites of berberine might be the main existing forms in the body which would account for the pharmacological effects in vivo [9]. Many studies have already demonstrated that the metabolites of berberine showed similar bio-activities to berberine [20,21,22]. For example, berberrubine exhibited hepatoprotective properties [23], hypolipidemic effects [24] and antitumor activities [25], etc. Demethyleneberberine showed hypolipidemic effects [26], hepatoprotective and anti-fibrotic effects [27], etc. Columbamine even exhibited more potential effects on TG-lowering and AMP-activated protein kinase (AMPK) activation in Hep G2 cells as compared with berberine [26]. Jatrorrhizine exhibited antioxidant activities [28], neuroprotective effects [29] and antitumor activities [30], etc. So, many pharmacological properties may derived from different metabolites which may generated by many different metabolic pathways. Thus, making clear the berberine metabolic pathways is of significant importance for elucidating its pharmacological mechanisms.

**Figure 5:**
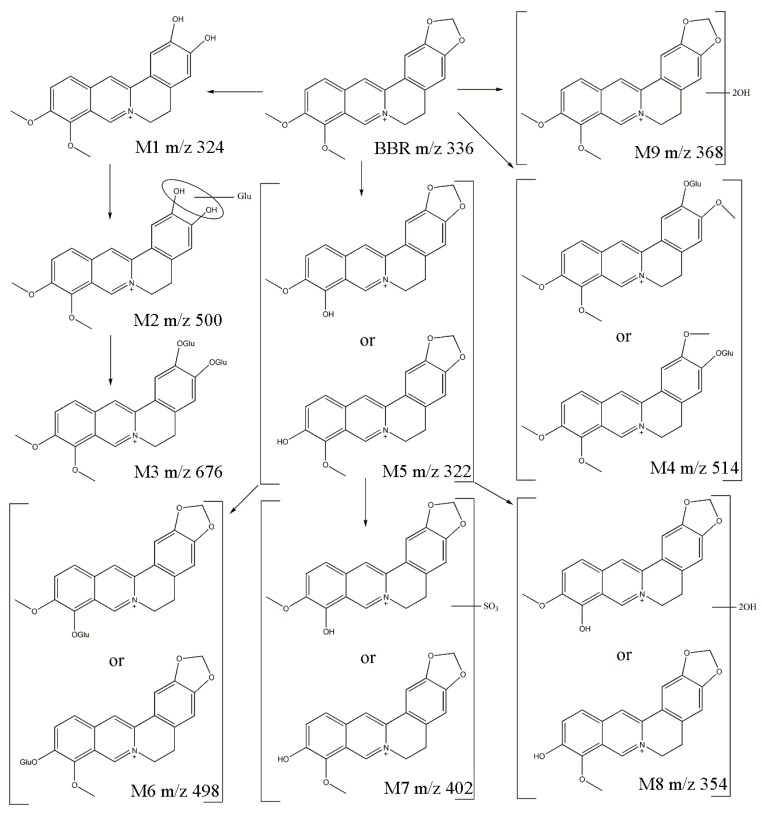
Structures of berberine metabolites and the possible metabolic pathways of berberine in rats.

Figure 5 shows the proposed metabolic pathway of berberine after administration of berberine in rats. Metabolites of Phase I were widely contained in rat plasma, including demethyleneberberine (M1), berberrubine or thalifendine (M5), dihydroxy berberrubine or thalifendine (M8) and dihydroxy berberine (M9). Conjugates with sulfite or glucuronic acid were also found. Metabolites of Phase II included glucuronic acid-conjugated demethyleneberberine (M2), diglucuronide-conjugated demethyleneberberine (M3), glucuronic acid-conjugated jatrorrhizine or columbamine (M4), glucuronic acid-conjugated berberrubine or thalifendine (M6) and sulfite-conjugated thalifendine or berberrubine (M7). In addition, dihydroxy berberrubine or thalifendine (M8), dihydroxy berberine (M9) and sulfite-conjugated berberrubine or thalifendine (M7) were found in rat plasma samples for the first time, which contributed to the finding of new metabolic pathways of berberine. Finally, the metabolism of berberine was elucidated comprehensively.

## 3. Materials and Methods

### 3.1. Chemicals

The reference standard of berberine was provided by the National Institute for Drug Control of China (Beijing, China). Ammonium acetate and formic acid of HPLC grade were purchased from Sigma-Aldrich (Shanghai, China). Acetonitrile (ACN) of HPLC grade was purchased from Merck (Darmstadt, Germany). Deionized water was prepared by Milli-Q system (Millipore, MA, USA).

### 3.2. Rat Studies

Male Sprague–Dawley (SD) rats with the weight of 180–220 g were purchased from the Experimental Animal Center of Fudan University. The rats were fed with standard laboratory food and water was provided ad libitum. The temperature of the animal room was maintained at 22 ± 2 °C and the relative humidity was maintained at 50 ± 10% with natural day/night cycle. Rats were fasted for 12 h before drug administration with free access to water. Berberine suspension (15 mg/mL berberine in 0.5% methylcellulose) was administered to rats by oral gavage at a dosage of 150 mg/kg. Blood samples of 400 μL were collected from the oculi choroid vein both before and 24 h subsequently following the administration, transferred to Eppendorf tubes containing heparin and then centrifuged at 4000 rpm for 5 min to obtain plasma samples. Then the supernatants were saved and stored at −20 °C for further analysis. All animal protocols were approved by the Fudan University Institutional Animal Care and Use Committee.

### 3.3. Sample Preparation

Acetonitrile of 300 μL was added to 100 μL rat plasma in a 1.5 mL Eppendorf tube. After vortex mixing for 1 min vigorously, the samples were centrifuged for 10 min at 10,000 rpm. The supernatant was transferred to clean Eppendorf tubes and evaporated to dryness at 40 °C under an N2 stream. The residue was then dissolved in 100 μL of 20% ACN and vortexed as the testing samples. An aliquot of 20 μL of the reconstituted samples was injected to the instrument for analysis.

### 3.4. Instrumental Parameters

The experiment was conducted on an Agilent series 1290 UHPLC system (Agilent, Germany) combined with a Q-TOF-MS/MS system which equipped with an ESI source (LC/MS Triple TOF^®^ 5600+, AB SCIEX, CA). The UHPLC instrument system included a diode-array detector, an autosampler, a binary pump and a column compartment. Samples were separated using an Agilent C18 RRHD column (2.1 × 50 mm, 1.8 μm). The temperature of the column compartment was adjusted to 30 °C. The mobile phase solutions were solvent A (0.1% of formic acid in water and 10 mM ammonium acetate) and solvent B (ACN) with linear gradient elution. The gradient elution program started by increasing the phase B from 5% to 100% in 12.0 min, and kept the composition during 12.0 to 15.0 min. After setting back to 5% phase B at 15.1 min, the mobile phase gradient elution program was maintained at the proportion from 15.1 to 18.0 min for column equilibration. The flow rate was set at 0.3 mL/min, and the injection volume of the sample was 20 μL.

Using a Triple TOF^®^ 5600+ mass spectrometer, MS and MS2 data were acquired through MS scan and information dependent acquisition MS2 scans by Analyst^®^ software. The mass spectrometer was performed in positive ion mode and the ESI source temperature was set at 550 °C. Collision gas and ion source gas were nitrogen. In the MS and MS2 experiments the instrumental conditions was optimized using acetonitrile solution of berberine in order to obtain the best parameters. The mass data acquisition was set in the range of *m/z* 100–1500 Da. The mass spectroscopic analysis was performed under the following conditions: ion source gas one was set at 50 psi and ion source gas two was 60 psi; curtain gas was 30 psi; ion spray voltage floating was set at 5500 V; collision energy was 40 V and declustering potential was 80 V. By information dependent acquisition MS2 scans, the MS2 data were obtained. Using a calibrated delivery system external mass calibration was performed automatically.

With the help of the PeakView 2.0 software, including extracted ion chromatograms and the chemical compositions calculation through potential metabolites, MS data processing was performed. The metabolites were automatically identified by MetabolitePilot^TM^ 1.5 software (AB Sciex, ON, Canada).

## 4. Conclusions

A reliable and fast analytical method for investigating the metabolism of berberine in rats was presented in this paper. In all, nine metabolites were tentatively characterized or identified. As far as we know, several new metabolic pathways and three metabolites in rat plasma are reported for the first time. The results of this paper are beneficial to further understanding of the metabolic profile of berberine in rats and the result of finding three new metabolites may serve as a complementary reference in the future for new pharmacological effects studies searching the metabolites of berberine.

## Figures and Tables

**Table 1:** Metabolites of berberine detected by UHPLC–ESI–Q–TOF–MS/MS in rat plasma.

Meta-Bolites	Formula	RT ^1^ (min)	*m/z* (Da)	Error (ppm)	Iso Diff ^2^ (%)	Fragments (*m/z*)	Identification
M1	C_19_H_18_NO_4_	2.79	324.1236	−1.4	0.3	308.0979	Demethylene-berberine
M2	C_25_H_26_NO_10_	2.78	500.1647	−0.1	0.3	324.1299 308.0977	glucuronic acid-conjugated M1
M3	C_31_H_34_NO_16_	2.23	676.1798	−0.8	2.6	500.1640 324.1292	diglucuronide-conjugated M1
M4	C_26_H_28_NO_10_	2.81	514.1805	−0.3	2.8	338.1459 322.1130	glucuronic acid-conjugated jatrorrhizine or columbamine
M5	C_19_H_16_NO_4_	3.72	322.1148	−1.7	4.7	307.0900 279.0944	berberrubine or thalifendine
M6	C_25_H_24_NO_10_	2.85	498.1479	−0.6	1.4	322.1138 307.0902	glucuronic acid-conjugated M5
M7	C_19_H_16_NO_7_S	3.82	402.0697	−0.9	2.6	322.1135 307.0908	sulfite-conjugat-ed M5
M8	C_19_H_16_NO_6_	3.90	354.0978	−1.0	2.3	308.1012 293.0726	dihydroxy M5
M9	C_20_H_18_NO_6_	5.43	368.1202	−0.8	3.2	322.1104 306.0809	Dihydroxy berberine

^1^ Retention time; ^2^ Isotope difference.

## References

[1] Karaosmanoglu K., Sayar N.A., Kurnaz I.A., Akbulut B.S. (2014). Assessment of berberine as a multi-target antimicrobial: A multi-omics study for drug discovery and repositioning. Omics.

[2] Zhang M., Long Y., Sun Y., Wang Y., Li Q., Wu H., Guo Z., Li Y., Niu Y., Li C. (2011). Evidence for the complementary and synergistic effects of the three-alkaloid combination regimen containing berberine, hypaconitine and skimmianine on the ulcerative colitis rats induced by trinitrobenzene-sulfonic acid. Eur. J. Pharmacol..

[3] Lau C.W., Yao X.Q., Chen Z.Y., Ko W.H., Huang Y. (2001). Cardiovascular actions of berberine. Cardiovasc Drug Rev..

[4] Shen Y.B., Piao X.S., Kim S.W., Wang L., Liu P. (2010). The effects of berberine on the magnitude of the acute inflammatory response induced by Escherichia coli lipopolysaccharide in broiler chickens. Poultry Sci..

[5] Wang N., Tan H., Li L., Yuen M., Feng Y. (2015). Berberine and Coptidis Rhizoma as potential anticancer agents: Recent updates and future perspectives. J. Ethnopharmacol..

[6] Doggrell S.A. (2005). Berberine - a novel approach to cholesterol lowering. Expert Opin. Investig. Drugs.

[7] Pirillo A., Catapano A.L. (2015). Berberine, a plant alkaloid with lipid- and glucose-lowering properties: From in vitro evidence to clinical studies. Atherosclerosis.

[8] Tsai P.L., Tsai T.H. (2002). HPLC determination of berberine in medicinal herbs and a related traditional Chinese medicine. Anal. Lett..

[9] Wang K., Feng X., Cha L., Cao S., Qiu F. (2017). The metabolism of berberine and its contribution to the pharmacological effects. Drug Metab. Rev..

[10] Ma J., Feng R., Tan X., Ma C., Shou J., Fu J., Huang M., He C., Chen S., Zhao Z. (2013). Excretion of Berberine and Its Metabolites in Oral Administration in Rats. J. Pharm. Sci..

[11] Pan J.F., Yu C., Zhu D.Y., Zhang H., Zeng J.F., Jiang S.H., Ren J.Y. (2002). Identification of three sulfite-conjugated metabolites of berberine chloride in healthy volunteers’ urine after oral administration. Acta Pharmacol. Sin..

[12] Tsai P.L., Tsai T.H. (2004). Hepatobiliary excretion of berberine. Drug Metab. Dispos..

[13] Tan X., Ma J., Feng R., Ma C., Chen W., Sun Y., Fu J., Huang M., He C., Shou J. (2013). Tissue Distribution of Berberine and Its Metabolites after Oral Administration in Rats. PLoS ONE.

[14] Li Y., Ren G., Wang Y., Kong W., Yang P., Wang Y., Li Y., Yi H., Li Z., Song D. (2011). Bioactivities of berberine metabolites after transformation through CYP450 isoenzymes. J. Trans. Med..

[15] Han X., Yin L., Xu L., Wang X., Peng J. (2010). Simultaneous determination of ten active components in chinese medicine “Huang-Lian-Shang-Qing” tablets by high-performance liquid chromatography coupled with photodiode array detection. Anal. Lett..

[16] Qi M., Xiong A., Li P., Yang Q., Yang L., Wang Z. (2013). Identification of acteoside and its major metabolites in rat urine by ultra-performance liquid chromatography combined with electrospray ionization quadrupole time-of-flight tandem mass spectrometry. J. Chromatogr. B.

[17] Li S., Liu W., Teng L., Cheng X., Wang Z., Wang C. (2014). Metabolites identification of harmane in vitro/in vivo in rats by ultra-performance liquid chromatography combined with electrospray ionization quadrupole time-of-flight tandem mass spectrometry. J. Pharmaceut. Biomed..

[18] Qiu F., Zhu Z., Kang N., Piao S., Qin G., Yao X. (2008). Isolation and Identification of Urinary Metabolites of Berberine in Rats and Humans. Drug Metab. Dispos..

[19] Zuo F., Nakamura N., Akao T., Hattori M. (2006). Pharmacokinetics of berberine and its main metabolites in conventional and pseudo germ-free rats determined by liquid chromatography/ion trap mass spectrometry. Drug Metab. Dispos..

[20] Spinozzi S., Colliva C., Camborata C., Roberti M., Ianni C., Neri F., Calvarese C., Lisotti A., Mazzella G., Roda A. (2014). Berberine and Its Metabolites: Relationship between Physicochemical Properties and Plasma Levels after Administration to Human Subjects. J. Nat. Prod..

[21] Porru E., Franco P., Calabria D., Spinozzi S., Roberti M., Caliceti C., Roda A. (2018). Combined analytical approaches to define biodistribution and biological activity of semi-synthetic berberrubine, the active metabolite of natural berberine. Anal. Bioanal. Chem..

[22] Zhou Y., Cao S., Wang Y., Xu P., Yan J., Bin W., Qiu F., Kang N. (2014). Berberine metabolites could induce low density lipoprotein receptor up-regulation to exert lipid-lowering effects in human hepatoma cells. Fitoterapia.

[23] Abd El-Salam M., Mekky H., El-Naggar E.M.B., Ghareeb D., El-Demellawy M., El-Fiky F. (2015). Hepatoprotective properties and biotransformation of berberine and berberrubine by cell suspension cultures of Dodonaea viscosa and Ocimum basilicum. S. Afr. J. Bot..

[24] Cao S., Xu P., Yan J., Liu H., Liu L., Cheng L., Qiu F., Kang N. (2019). Berberrubine and its analog, hydroxypropyl-berberrubine, regulate LDLR and PCSK9 expression via the ERK signal pathway to exert cholesterol-lowering effects in human hepatoma HepG2 cells. J. Cell Biochem..

[25] Li X., Li C., Xiao J., Gao H., Wang H., Zhang X., Zhang C., Ji X. (2015). Berberine Attenuates Vascular Remodeling and Inflammation in a Rat Model of Metabolic Syndrome. Biol. Pharm. Bull..

[26] Cao S., Zhou Y., Xu P., Wang Y., Yan J., Bin W., Qiu F., Kang N. (2013). Berberine metabolites exhibit triglyceride-lowering effects via activation of AMP-activated protein kinase in Hep G2 cells. J. Ethnopharmacol..

[27] Wang Y., Zhao Z., Yan Y., Qiang X., Zhou C., Li R., Chen H., Zhang Y. (2016). Demethyleneberberine Protects against Hepatic Fibrosis in Mice by Modulating NF-κB Signaling. Int. J. Mol. Sci..

[28] Rackova L., Majekova M., Kost’alova D., Stefek M. (2004). Antiradical and antioxidant activities of alkaloids isolated from Mahonia aquifolium. Structural aspects. Bioorg. Med. Chem..

[29] Luo T., Jiang W., Kong Y., Li S., He F., Xu J., Wang H.Q. (2016). The protective effects of jatrorrhizine on b-amyloid (25-35)-induced neurotoxicity in rat cortical neurons. CNS Neurol Disord. Drug Targets.

[30] Liu R.F., Cao Z.F., Pan Y.Y., Zhang G.C., Yang P., Guo P.D., Zhou Q.S. (2013). Jatrorrhizine hydrochloride inhibits the proliferation and neovascularization of C8161 metastatic melanoma cells. Anticancer Drugs.

